# Serum metabolites and hypercholesterolemia: insights from a two-sample Mendelian randomization study

**DOI:** 10.3389/fcvm.2024.1410006

**Published:** 2024-07-25

**Authors:** Weitao Wang, Jingwen Qiao, Zhaoyin Su, Hui Wei, Jincan Wu, Yatao Liu, Rubing Lin, Nerich Michael

**Affiliations:** ^1^ The First Clinical College of Medicine, Lanzhou University, Lanzhou, China; ^2^ Graduate Department of Shanxi Medical University, Taiyuan, Shanxi, China; ^3^ School of Stomatology, Lanzhou University, Lanzhou, China; ^4^ The Second Affiliated Hospital of Fujian University of Traditional Chinese Medicine, Fuzhou, China; ^5^ Department of Anesthesia, First Hospital of Lanzhou University, Lanzhou, China; ^6^ Department of Orthopedics, Shenzhen Children’s Hospital, Shenzhen, Guangdong, China; ^7^ Department of Trauma Surgery, University Hospital Regensburg, Regensburg, Germany

**Keywords:** Mendelian randomization, causality, hypercholesterolemia, metabolites, treatment

## Abstract

**Background:**

Hypercholesterolemia, a critical contributor to cardiovascular disease, is not fully understood in terms of its relationship with serum metabolites and their role in disease pathogenesis.

**Methods:**

This study leveraged GWAS data to explore the relationship between serum metabolites and hypercholesterolemia, pinpointing significant metabolites via Mendelian Randomization (MR) and KEGG pathway enrichment analysis. Data on metabolites were sourced from a European population, with analysis focusing on individuals diagnosed with hypercholesterolemia.

**Results:**

Out of 486 metabolites analyzed, ten showed significant associations with hypercholesterolemia, categorized into those enhancing risk and those with protective effects. Specifically, 2-methoxyacetaminophen sulfate and 1-oleoylglycerol (1-monoolein) were identified as risk-enhancing, with odds ratios (OR) of 1.545 (95% CI: 1.230–1.939; P_FDR = 3E−04) and 1.462 (95% CI: 1.036–2.063; P_FDR = 0.037), respectively. On the protective side, 3-(cystein-S-yl)acetaminophen, hydroquinone sulfate, and 2-hydroxyacetaminophen sulfate demonstrated ORs of 0.793 (95% CI: 0.735–0.856; P_FDR = 6.18E−09), 0.641 (95% CI: 0.423–0.971; P_FDR = 0.042), and 0.607 (95% CI: 0.541–0.681; P_FDR = 5.39E−17), respectively. In addition, KEGG pathway enrichment analysis further revealed eight critical pathways, comprising “biosynthesis of valine, leucine, and isoleucine”, “phenylalanine metabolism”, and “pyruvate metabolism”, emphasizing their significant role in the pathogenesis of hypercholesterolemia.

**Conclusion:**

This study underscores the potential causal links between particular serum metabolites and hypercholesterolemia, offering innovative viewpoints on the metabolic basis of the disease. The identified metabolites and pathways offer promising targets for therapeutic intervention and warrant further investigation.

## Introduction

Cardiovascular diseases (CVD) incur global losses exceeding $863 billion, imposing a substantial economic burden projected to reach $1, 044 billion by 2030 (1), thereby exacerbating the global disease burden. Hypercholesterolemia, a key factor in CVD, is marked by elevated levels of total cholesterol (TC) and low-density lipoprotein cholesterol (LDL-C) in the bloodstream. Prolonged duration of elevated LDL-C levels markedly heightens the potential for early-onset atherosclerotic cardiovascular diseases and increases the likelihood of premature mortality (2). Hypercholesterolemia represents a critical health concern, established as a primary cause of early-onset myocardial infarction (3). Prompt diagnosis and efficacious lipid-lowering therapy are essential for these patients (3–8).

Hypercholesterolemia is a metabolic disorder, and despite significant advancements in its treatment over the past two decades (9), its exact pathophysiology and specific biomarkers are still not well understood (10). Previous studies have suggested potential risk factors such as gut microbiota and cytokines (11–14), but research focusing on the overall metabolic alterations in hypercholesterolemia is relatively understudied.

In recent years, the field of metabolomics has garnered substantial attention within systems biology, offering essential understanding of the detailed biological processes behind various diseases, such as hypercholesterolemia (15, 16). Metabolomics represents a cutting-edge and efficient analytical approach that facilitates the concurrent quantification of thousands of small molecules within biological specimens (17). Located downstream of the genome, metabolomics offers essential insights into the dynamics of intricate biological systems. It is applied across a broad spectrum of health conditions, providing new perspectives on pathogenic processes and aiding in the identification of biomarkers for detection and treatment (18). Consequently, metabolomics promotes a comprehensive biological description of organisms (19, 20). The advancement of human genome sequencing technology has pioneered an innovative period of customized healthcare, in which genetic variations can forecast the effectiveness of specific therapeutic strategies, optimizing individual disease management (21).

Additionally, the targeted modulation of metabolites holds considerable promise for managing hypercholesterolemia. For instance, bile acids have been extensively studied. Bile acid sequestrants work by decreasing the enterohepatic recirculation of bile acids, resulting in a decrease of the bile acid pool by approximately 40%. This reduction stimulates the transformation of cholesterol into bile acids (cholesterol catabolism) and increases the elimination of bile acids through fecal matter (22–24). Consequently, the stimulation of cholesterol conversion to bile acids helps lower blood lipid levels. Therefore, the targeted metabolic regulation of bile acids emerges as an encouraging therapeutic approach for managing hypercholesterolemia (25).

Investigating metabolites linked to the initiation and development of hypercholesterolemia is essential not just for early detection and prevention but also essential for understanding the biological mechanisms underlying the treatment of hypercholesterolemia. Earlier research has explored the relationship between specific metabolites and diseases using metabolomic approaches (15, 16). Nonetheless, the causal link between metabolites and hypercholesterolemia is still ambiguous owing to the limited number of prospective studies on this topic. Conventional observational studies face design limitations, such as lifestyle modifications or long-term medication after the diagnosis of hypercholesterolemia, which can alter metabolite levels, thereby obscuring the causal link between metabolites and hypercholesterolemia. Despite being the premier method for establishing causality, randomized controlled trials present substantial challenges in this area, making it difficult to ascertain definitive conclusions on the causative link between metabolites and hypercholesterolemia.

Due to the various limitations of randomized controlled trials, Mendelian randomization has emerged as an effective alternative for inferring causal relationships (26). MR employs single nucleotide polymorphisms (SNPs) as proxies for exposure factors to robustly assess the causal relationship between exposures and outcomes (27). This approach simulates the random allocation in randomized controlled trials, as SNPs are randomly distributed in the population, thereby reducing the impact of confounding factors such as sex on the outcomes. Moreover, since genotypes are determined before disease onset, MR minimizes the risk of reverse causation (28).

Given the limited research on the relationship between blood metabolites and hypercholesterolemia, further studies are urgently needed. This study utilizes genome-wide association studies (GWAS) data for MR analysis to infer the causal relationship between 486 blood metabolites and hypercholesterolemia.

## Materials and methods

**Figure 1:**
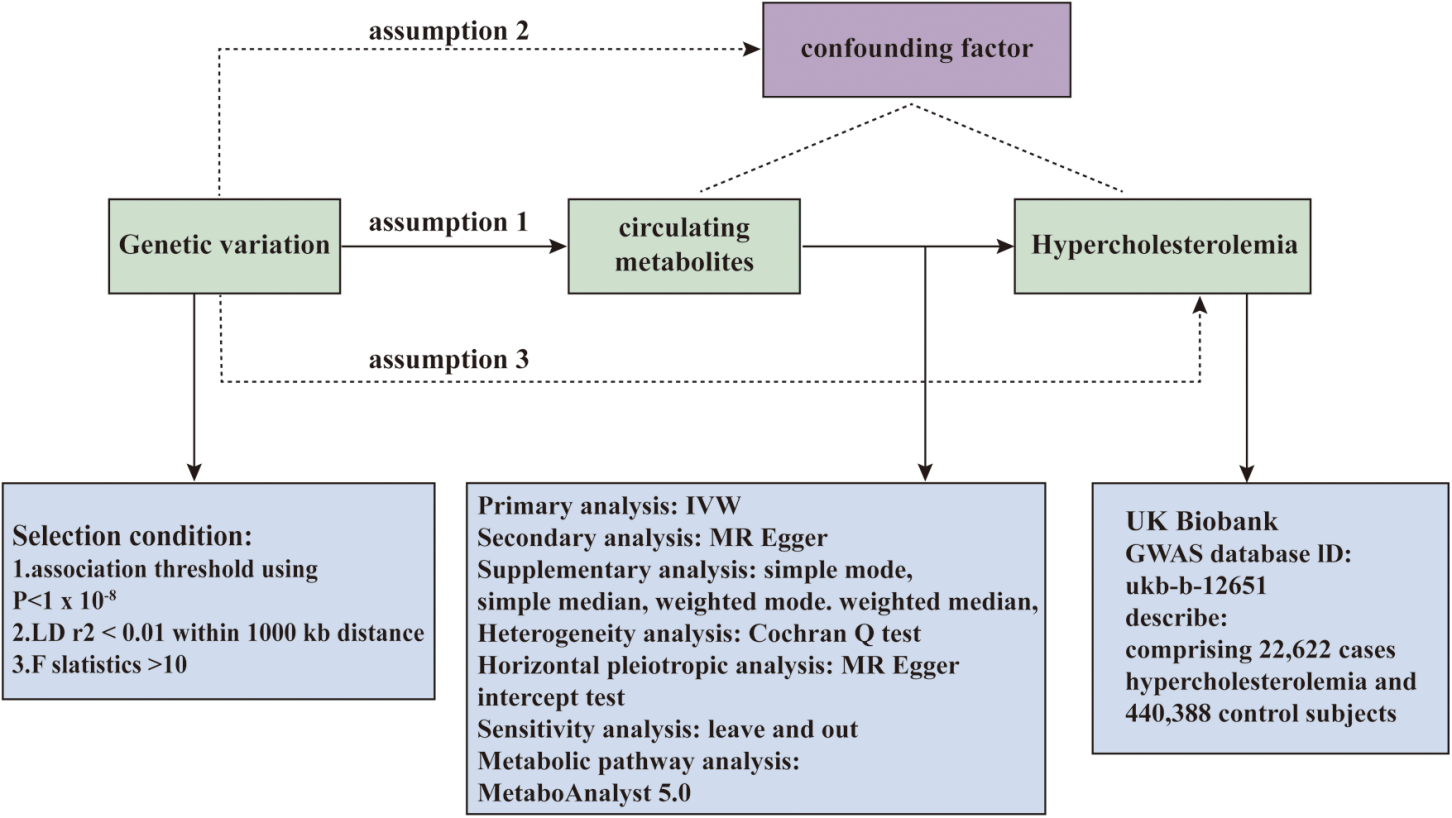
The overall designation of this study.

Our study employs a two-sample Mendelian Randomization approach to rigorously assess the causative relationship between metabolites identified in individual blood samples and the risk of onset of hypercholesterolemia. An effective MR study necessitates meeting the following assumptions: 1. The Genetic instruments must be strongly correlated with the exposure; 2. Genetic instruments should remain unlinked to potential confounders; 3. Genetic instruments must influence the outcome solely through the exposure. Independence from horizontal pleiotropy, relevant to the second and third assumptions, can be assessed through a range of statistical techniques. We acquired data on hypercholesterolemia from the UK Biobank GWAS consortium, as outlined in Figure 1, offering a summary of the study's design.

### Data source

The GWAS data on blood metabolites are derived from the study by Shin et al. (29), which included extensive metabolomic analyses. The investigation included 7,824 participants of European descent, analyzing approximately 2.1 million SNPs and encompassing 486 metabolites. Based on the KEGG database, we identified 278 metabolites with specific chemical characteristics and classified them into eight metabolic groups (30). The data for hypercholesterolemia were retrieved from the UK Biobank dataset, which included 463,010 participants. This includes 22,622 individuals diagnosed with hypercholesterolemia and 440,388 control subjects, all of whom are of European descent.

### Instrument selection

In our study, we employed various strategies to identify SNPs associated with the exposure (metabolites). Due to the lack of significant associations, we adjusted the threshold to *P* < 1 × 10^−5. Additionally, we utilized pair-wise linkage disequilibrium (LD) pruning within a 10,000 kb range (r^2 < 0.001) to identify the top independent SNPs. Furthermore, SNPs associated with hypercholesterolemia or confounding factors such as sex, BMI, obesity, diabetes, hypertension, smoking and medication use were excluded using the Phenoscanner database (31). The SNPs ultimately used for MR analysis are detailed in Supplementary Table S4 of Supplementary File 1. This commonly used methodology has been applied in prior MR studies. In parallel, we evaluated the F-statistic for each SNP to evaluate its statistical robustness, thereby reducing bias from weak instruments. To guarantee all SNPs generated adequate variance for their associated metabolites, we removed weak instruments with an F-statistic below 10. We also eliminated SNPs that were missing or lacked suitable proxies. Ultimately, metabolites containing a minimum of three SNPs were chosen for further analysis.

### MR analysis

The inverse variance weighting (IVW) method, is recognized as one of the most reliable techniques for MR estimation, despite its susceptibility to pleiotropy bias. In our research, we employed IVW with random effects as the primary method for MR analysis, adjusting the significance threshold to *P* < 0.05, to initially explore the association between metabolites and disease risk (hypercholesterolemia). Additionally, we employed five other MR analysis methods for examining our study outcomes. These MR analytical techniques were utilized to perform sensitivity analyses of our research outcomes.

### Statistical analysis

Statistical analysis employed the “TwoSampleMR” package (version 0.5.7) within R software (version 4.3.1). Of the six MR methods detailed previously, we designated the results from the IVW method as the main MR estimate and assessed their consistency with outcomes from the other MR approaches. Finally, a leave-one-out analysis was implemented to identify any disproportionate impact of specific SNPs on the results of each MR study.

### Visualization of the results

The conclusive findings are illustrated with a volcano plot, which succinctly represents the data by correlating the magnitude of effect with statistical significance. This graphical approach efficiently highlights metabolites of substantial impact on disease risk, delineating both the strength and reliability of their associations. For metabolites demonstrating significant causal relationships, forest plots are deployed. These graphical representations meticulously detail the effect sizes and confidence intervals for each metabolite, providing a precise comprehension of their relative significance and the confidence in these effects on disease risk.

### Metabolic pathway analysis

Further examination of metabolites with notable differences involves KEGG pathway enrichment analysis. This analysis is supported by MetaboAnalyst 5.0, which examines the metabolic pathways of key circulating metabolites. This platform integrates bioinformatic data and computational techniques, facilitating comprehensive characterization of the biological roles of a diverse set of genes and proteins. Results with a *P*-value below 0.05 are considered statistically significant, whereas those with a *P*-value ranging from 0.05 to 0.10 are deemed to have potential statistical significance.

## Results

Table 1 presents the GWAS data sources for this study. Following suitable screening, we selected 278 metabolites out of 486 for MR analysis; the other 208 metabolites were not identified (Supplementary File 1 includes Supplementary Table S1: A summary of causal effect estimates obtained through diverse MR methodologies; Supplementary Table S2: A summary of heterogeneity analysis results obtained through diverse MR methodologies; Supplementary Table S3: A summary of pleiotropy assessments obtained through diverse MR methodologies; Supplementary Table S4: A summary of the raw data; Supplementary Table S5: Detailed summaries of causal effect estimates for significant metabolites calculated using various MR methods. The SNP count per metabolite varied between 4 and 257. An important observation was that the F-statistics for all SNPs exceeded 10, suggesting that all instrumental variables were sufficiently strong.

**Table 1:** Overview of GWAS data covered in this research.

Phenotype	GWAS data source	Cohort	Size	Race
Human blood metabolites	Shin et al., 2014	European population studies	7,824	European
Hypercholesterolemia	Finn-b-DM_RETINOPATHY	UK Biobank	22,622 cases 440,388 controls	European

### The causal impact of circulating metabolites on hypercholesterolemia

**Figure 2:**
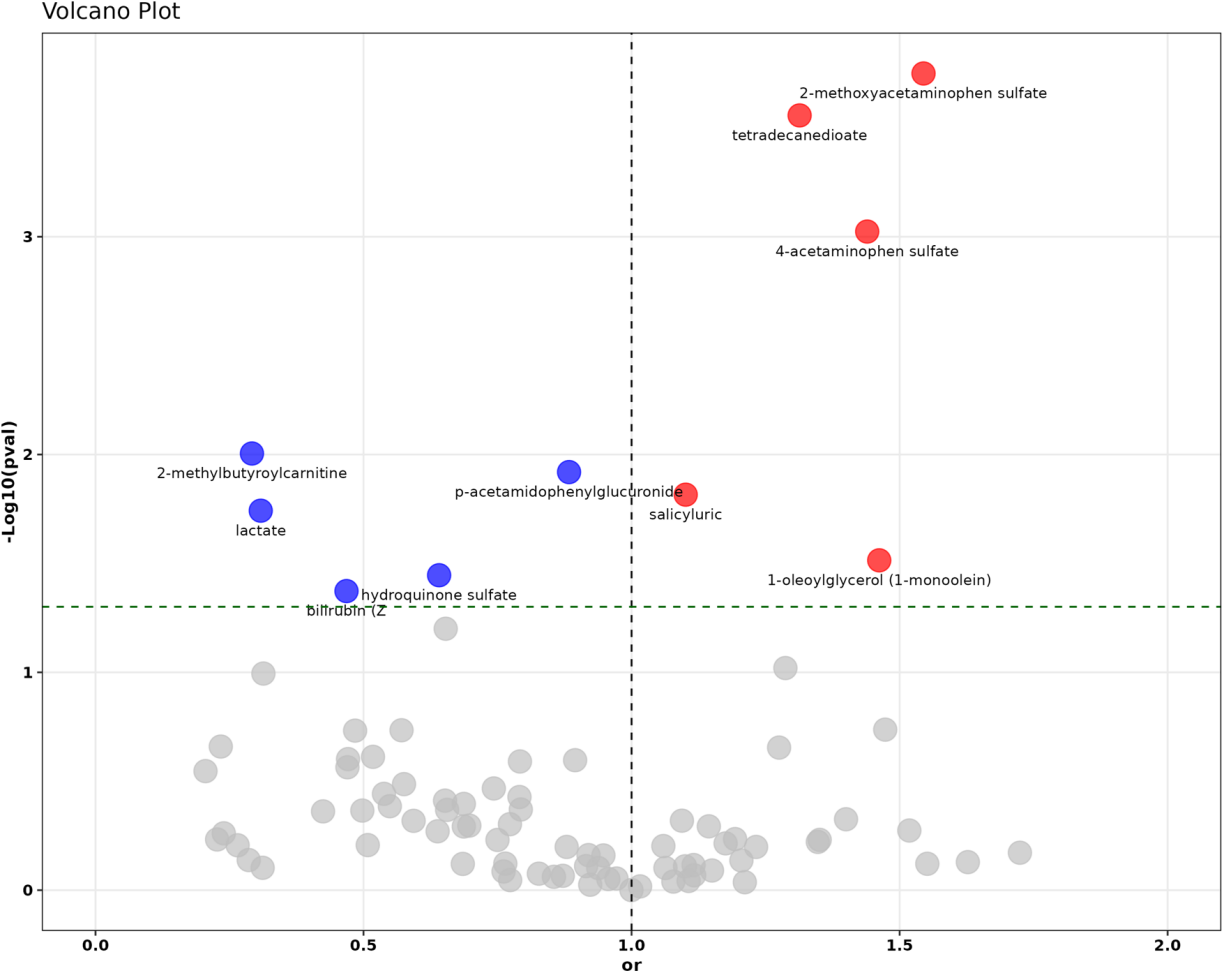
The volcano plot illustrates the causal relationship assessment and statistical significance of 278 circulating metabolites with hypercholesterolemia. Red dots represent statistically significant risk factors (OR >1), blue dots indicate statistically significant protective factors (OR <1), and gray dots denote metabolites without statistical significance.

In the investigation of the identified 278 metabolites through MR using the IVW method, 65 metabolites showed significant associations with Disease Risk (hypercholesterolemia) as illustrated in Figure 2 and detailed in Supplementary Table S5. Nonetheless, following numerous corrections for the False Discovery Rate (FDR) and comprehensive consideration, only ten metabolites remained significantly associated with hypercholesterolemia. Among these, 2-methoxyacetaminophen sulfate demonstrated an Odds Ratio (OR) per SD increase of 1.545 (95% CI: 1.230–1.939; *P* = 1.78E−04, P_FDR = 3E−04), while 1-oleoylglycerol (1-monoolein) had an OR per SD increase of 1.462 (95% CI: 1.036–2.063; *P* = 0.031, P_FDR = 0.037), indicating a significant correlation with a heightened risk of hypercholesterolemia.

**Figure 3:**
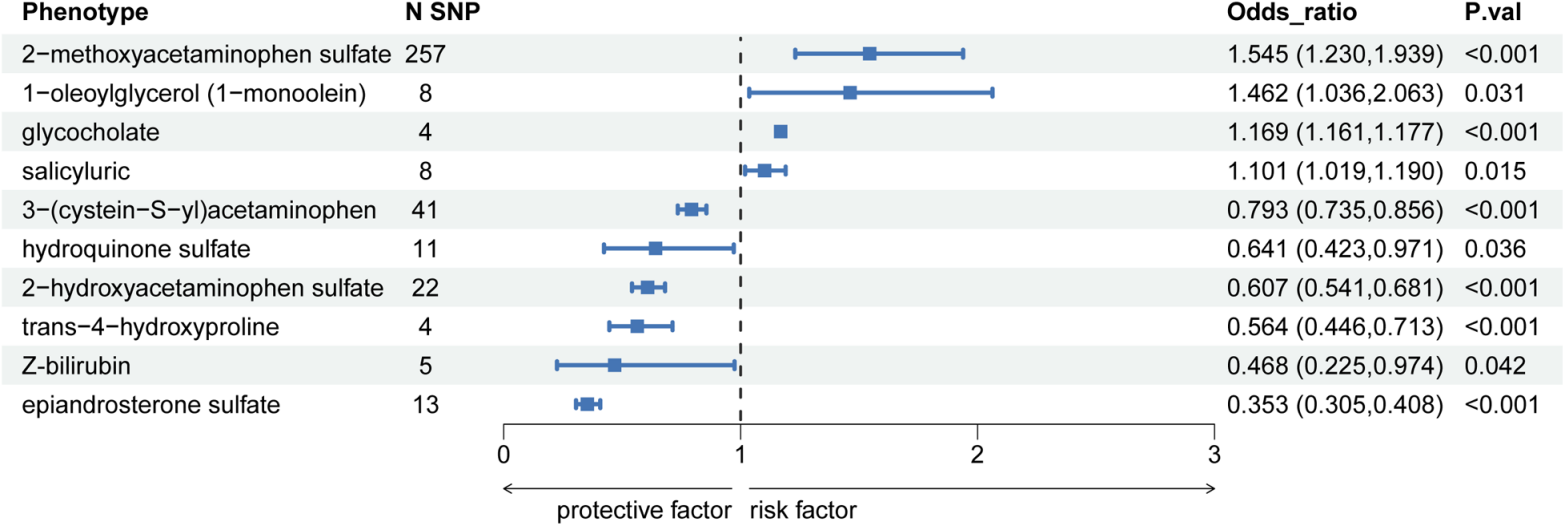
Forest diagram of causal correlation between blood metabolites and hypercholesterolemia.

Conversely, metabolites such as 3-(cystein-S-yl)acetaminophen exhibited an OR per SD increase of 0.793 (95% CI: 0.735–0.856; *P* = 2.39E-09, P_FDR = 6.18E-09), hydroquinone sulfate had an OR per SD increase of 0.641 (95% CI: 0.423–0.971; *P* = 0.035, P_FDR = 0.042), and epiandrosterone sulfate showed an OR per SD increase of 0.353 (95% CI: 0.305–0.400; *P* = 2.64E-45, P_FDR = 1.63E-44), demonstrated significant causal relationships with hypercholesterolemia, potentially offering protective effects. The detailed findings are presented in Figure 3, with additional information available in Supplementary Table S1.

### Evaluation of heterogeneity and horizontal pleiotropy

In the assessment of heterogeneity related to 10 circulating metabolites significantly associated with hypercholesterolemia, heterogeneity was observed in 2-methoxyacetaminophen sulfate, 1-oleoylglycerol (1-monoolein), 3-(cystein-S-yl)acetaminophen, 2-hydroxyacetaminophen sulfate, and epiandrosterone sulfate. No evidence of heterogeneity was found in the additional circulating metabolites. Supplementary Table S2 provides the complete outcomes. Pleiotropy analysis showed that the effects of metabolites on hypercholesterolemia causality were not influenced by horizontal pleiotropy at the SNP level. The specific findings are presented in Supplementary Table S3. Additionally, findings from the LOO analysis verified that the MR estimates of individual SNPs were unbiased, as illustrated in Supplementary Figures S1–S10.

### Metabolic pathway analysis

**Figure 4:**
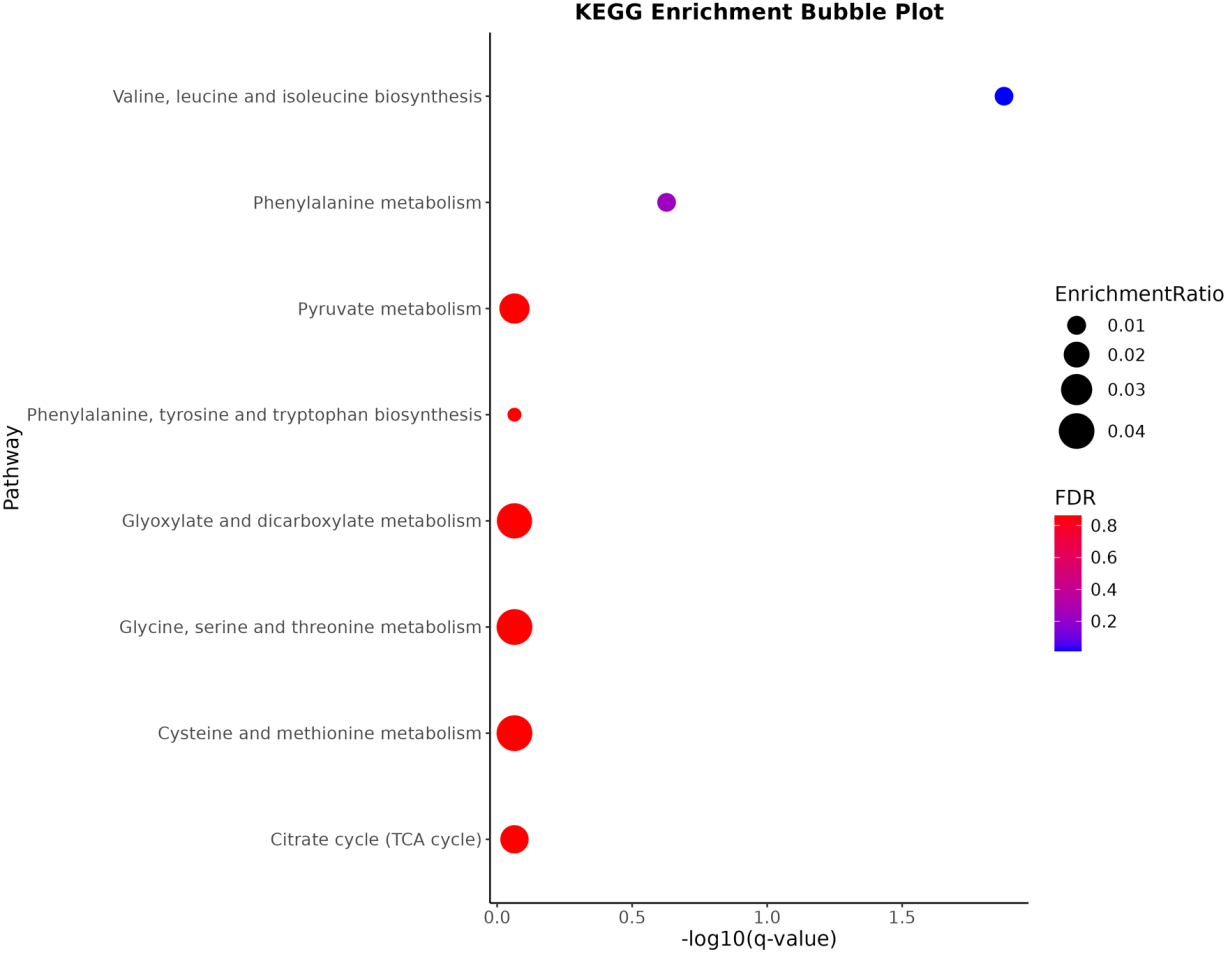
The top 8 genomes-enriched pathways.

During the KEGG pathway enrichment analysis, significant changes in 74 circulating metabolites were assessed, leading to the identification of eight potential metabolic pathways linked to hypercholesterolemia. Of these pathways, the three most crucial were “Valine, leucine and isoleucine biosynthesis, “Phenylalanine metabolism,” and “Pyruvate metabolism,” with *P*-values of 0.00016584, 0.0058945, and 0.046341 (all *P* < 0.05). The fourth key pathway identified was also “Phenylalanine metabolism, “ playing a vital part in the context of hypercholesterolemia, as depicted in Figure 4.

## Discussion

In this study, utilizing GWAS data, we identified 10 out of 486 blood metabolites linked to hypercholesterolemia. Of the identified metabolites, four metabolites exhibited harmful effects related to an increased risk of hypercholesterolemia, including 2-methoxyacetaminophen sulfate, 1-oleoylglycerol (1-monoolein), glycocholate, and salicyluric, while six metabolites showed protective effects against hypercholesterolemia, namely 3-(cystein-S-yl)acetaminophen, hydroquinone sulfate, 2-hydroxyacetaminophen sulfate, trans-4-hydroxyproline, Z-bilirubin, and epiandrosterone sulfate. Various analytical methods further validated these findings.

Hypercholesterolemia is a metabolic disorder marked by increased levels of TC and LDL-C in the blood. This condition adversely affects the cardiovascular system and can trigger cardiovascular diseases (2). While genetic, nutritional, and lifestyle factors contribute to the progression of hypercholesterolemia, its precise mechanisms remain not fully elucidated (32–34). Early diagnosis and intervention are crucial for preventing cardiovascular events, highlighting the necessity of reliable biomarkers to optimize therapeutic strategies for hypercholesterolemia.

Metabolomics has garnered widespread attention for its potential to identify metabolites associated with hypercholesterolemia. Blood metabolites can reveal both endogenous and exogenous metabolic activities, offering valuable insights into disease mechanisms. For example, Haihua Bai et al. utilized LC/MS for serum analysis of healthy controls and hypercholesterolemia patients, revealing significant differences in serum metabolites between these groups (15). Additionally, numerous studies have reported increased oxidative stress in hypercholesterolemia patients, particularly a reduction in the cellular antioxidant glutathione (35). Considering that the metabolomic alterations in blood mirror intracellular changes, it is crucial to precisely study metabolic regulation in different cell types of hypercholesterolemia patients. While the association between metabolites and hypercholesterolemia has been preliminarily established, further research is required to determine a definitive causal relationship and to gain a deeper insight into the fundamental mechanisms (16). These examinations could broaden our comprehension of hypercholesterolemia's development and may aid in devising robust strategies for early identification and prevention. Hence, we conducted this study to elucidate the causal link between blood metabolites and hypercholesterolemia, providing novel approaches for the screening and management of this condition.

In our study, we identified that genetic predispositions to four blood metabolites [2-methoxyacetaminophen sulfate, 1-oleoylglycerol (1-monoolein), glycocholate, and salicyluric] are linked to an increased risk of hypercholesterolemia. Despite limited reports discussing their impacts, some insights suggest further investigation is warranted. For instance, 2-methoxyacetaminophen sulfate, an acetamide compound, acts as a drug metabolite. Historically, acetamides have been considered negatively correlated with levels of glutathione and N-acetylcysteine (NAC) (36). NAC has been used to prevent cardiovascular conditions like coronary artery disease and myocardial infarction, primarily due to its antioxidant properties (37). Research has demonstrated that NAC pre-treatment can markedly suppress the conversion of mouse 3T3-L1 preadipocytes to adipocytes, thereby reducing intracellular lipid buildup and the expression of proteins related to obesity (38), indicating its important role in lipoprotein metabolism. Nevertheless, the precise roles and mechanisms of 2-methoxyacetaminophen sulfate are still not fully understood and require additional investigation. Notably, glycocholate, a bile acid, is associated with cardiovascular diseases. In the human body, bile acids are primarily synthesized from cholesterol (39). Substantial evidence links alterations in bile acid metabolism to cardiovascular diseases (40). Research indicates that bile acids can activate multiple signaling pathways, leading to elevated levels of total cholesterol, VLDL, and LDL, while reducing HDL levels (41–43). Currently, bile acid sequestrants targeting bile acids have been clinically used to treat hypercholesterolemia and have shown promising efficacy (25, 44).

Furthermore, our study identified that several metabolites, including 3-(cystein-S-yl)acetaminophen, hydroquinone sulfate, 2-hydroxyacetaminophen sulfate, trans-4-hydroxyproline, Z-bilirubin, and epiandrosterone sulfate, might have protective effects against hypercholesterolemia. Both 2-hydroxyacetaminophen sulfate and 3-(cystein-S-yl)acetaminophen are acetaminophen metabolites, and current research primarily focuses on their roles in liver injury (45, 46). Liver function impairment is associated with alterations in circulating lipids (47, 48), often manifesting as reduced levels of plasma cholesterol, lipoproteins, and triglycerides (49). Several observations support that patients with cirrhosis have significantly lower serum total cholesterol and TG levels compared to healthy individuals, possibly due to liver damage influencing clinical manifestations, including bacterial infections, hematological complications, malnutrition, and adrenal insufficiency (50). We speculate that the protective effects of 2-hydroxyacetaminophen sulfate and 3-(cystein-S-yl) acetaminophen on hypercholesterolemia may be mediated through liver injury mechanisms. Z-bilirubin, an isomer of bilirubin, forms as an ultimate product of heme catabolism (51). Studies over the past decade have shown that moderately elevated bilirubin levels can prevent metabolic and cardiovascular diseases (52–54). Studies have demonstrated that bilirubin treatment lowers total cholesterol levels in diet-induced obese mice (55), possibly by enhancing the ubiquitination and subsequent degradation of HMGCR, a key enzyme in cholesterol synthesis (56). Additionally, bilirubin alleviates oxidative stress by modulating Th17 immune responses and suppressing Toll-like receptor 4-induced ROS production (57, 58). Therapies targeting bilirubin have shown promising results in conditions such as pancreatitis, cardiac ischemia-reperfusion injury (IRI), and atherosclerosis (59, 60), suggesting potential efficacy for treating hypercholesterolemia. Epiandrosterone sulfate, a metabolite of dehydroepiandrosterone (DHEA), has been inversely associated with atherosclerosis in clinical studies (61). However, literature on the effects of epiandrosterone sulfate is limited, and its relationship with hypercholesterolemia warrants further investigation.

Furthermore, KEGG pathway enrichment analysis revealed eight significant metabolic pathways associated with hypercholesterolemia. These pathways include “Valine, leucine and isoleucine biosynthesis,” “Phenylalanine metabolism,” and “Pyruvate metabolism.” Notably, leucine, isoleucine, and valine have been significantly associated with atherosclerosis and are considered surrogate markers for this condition (62, 63). The valine metabolite, 3-hydroxyisobutyric acid, can stimulate trans-endothelial transport of fatty acids, resulting in localized lipid buildup and lipotoxicity (64). Additionally, BCAAs can disrupt mitochondrial pyruvate utilization by inhibiting the pyruvate dehydrogenase complex and reducing glucose oxidation (65), potentially causing the body to rely more on fatty acids and resulting in lipid metabolism disorders (66). Additionally, elevated phenylalanine levels can impact HMGR activity, subsequently inhibiting cholesterol synthesis (67). Pyruvate metabolism is essential to cholesterol production, functioning as a precursor for acetyl-coenzyme A, which is critical for cholesterol synthesis (68, 69). These metabolic disruptions may contribute to the development of hypercholesterolemia.

This MR analysis has several notable strengths. To our knowledge, this study is distinguished by its comprehensive examination of 486 blood metabolites and their possible effects on hypercholesterolemia. We applied stringent methodologies to mitigate potential issues and ensure the robustness of our findings. Various techniques were implemented to appropriately handle and exclude factors that violate MR assumptions. The study is committed to generating reliable estimates, as demonstrated by our careful management of potential confounders. Through the use of rigorous methodologies and the consideration of multiple variables, the researchers successfully produced dependable findings. Sensitivity analyses further validated our outcomes across different conditions and assumptions.

Our current research faces several challenges. First, Due to the categorization of the initial dataset, we were unable to evaluate the severity of hypercholesterolemia and had to analyze the condition in its entirety. Second, the analysis focused exclusively on individuals of European descent, thus our findings may not be generalizable to other populations; hence, subsequent research should seek to validate the metabolites identified in populations beyond European subjects to confirm the universality of our findings. Third, although our study has inferred associations between multiple metabolites and hypercholesterolemia, additional studies are required to elucidate the specific functions of these metabolites in the development of hypercholesterolemia.

Among these identified metabolites, Z-bilirubin has shown effective action against hypercholesterolemia and may offer a potential avenue for therapy. This study also revealed several metabolic pathways potentially associated with the development of hypercholesterolemia. These metabolites and their corresponding pathways could prove valuable in a medical setting for the early identification and prevention of hypercholesterolemia. These metabolites also hold promise as emerging treatment targets for hypercholesterolemia in the future.

## Data Availability

The original contributions presented in the study are included in the article/Supplementary Material, further inquiries can be directed to the corresponding author.
