# Supplementary figures and images for: Serum metabolites and hypercholesterolemia: insights from a two-sample Mendelian randomization study
Source: Front Cardiovasc Med. 2024 Jul 25;11:1410006. doi: 10.3389/fcvm.2024.1410006 (PMC11337230; doi:10.3389/fcvm.2024.1410006)

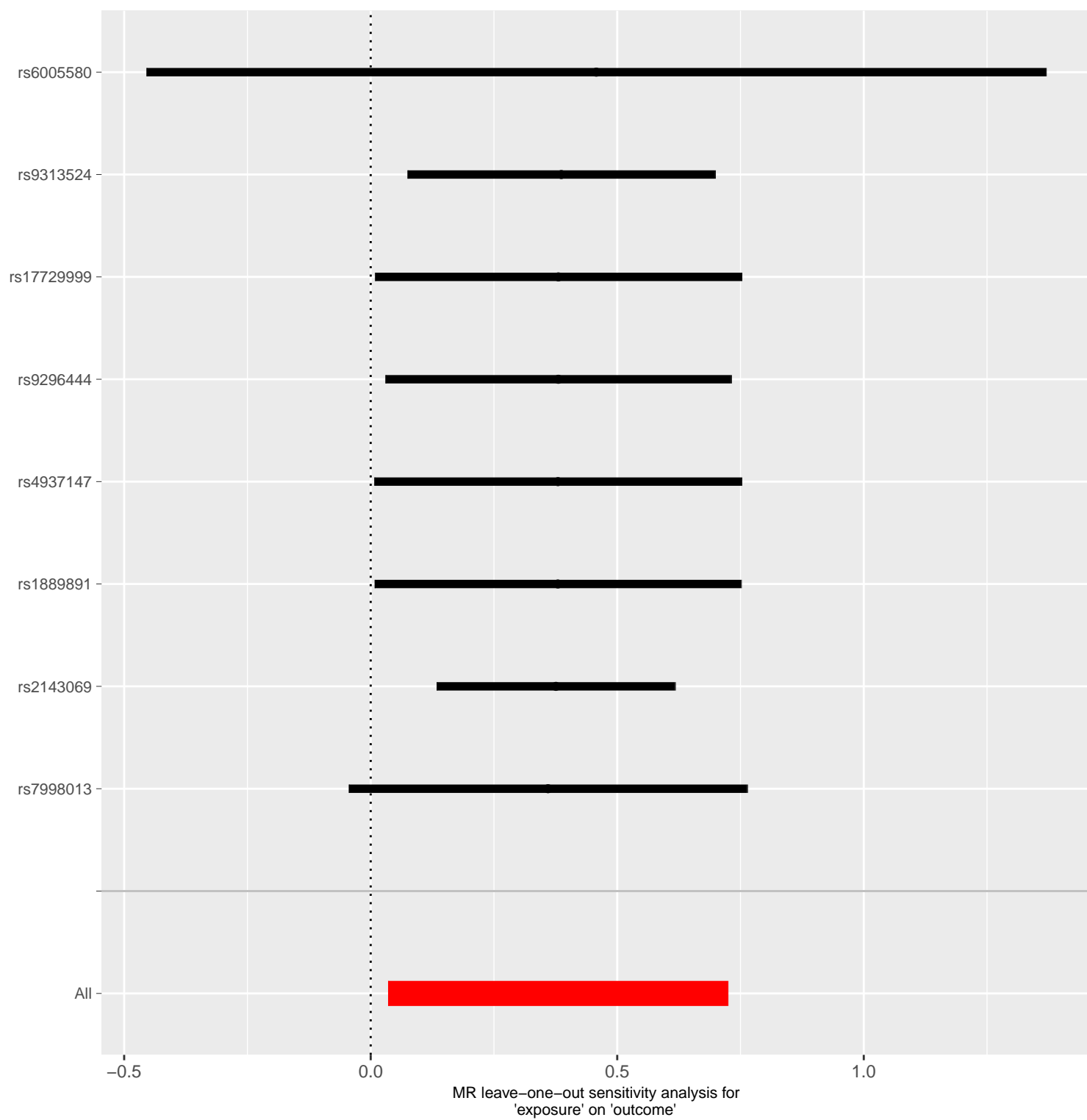

Supplement: Supplementary file 2 [file Datasheet1.zip › Data Sheet 1_v1/Supplementary Figures 1-10/1-oleoylglycerol (1-monoolein)_sensitivity-analysis.pdf]

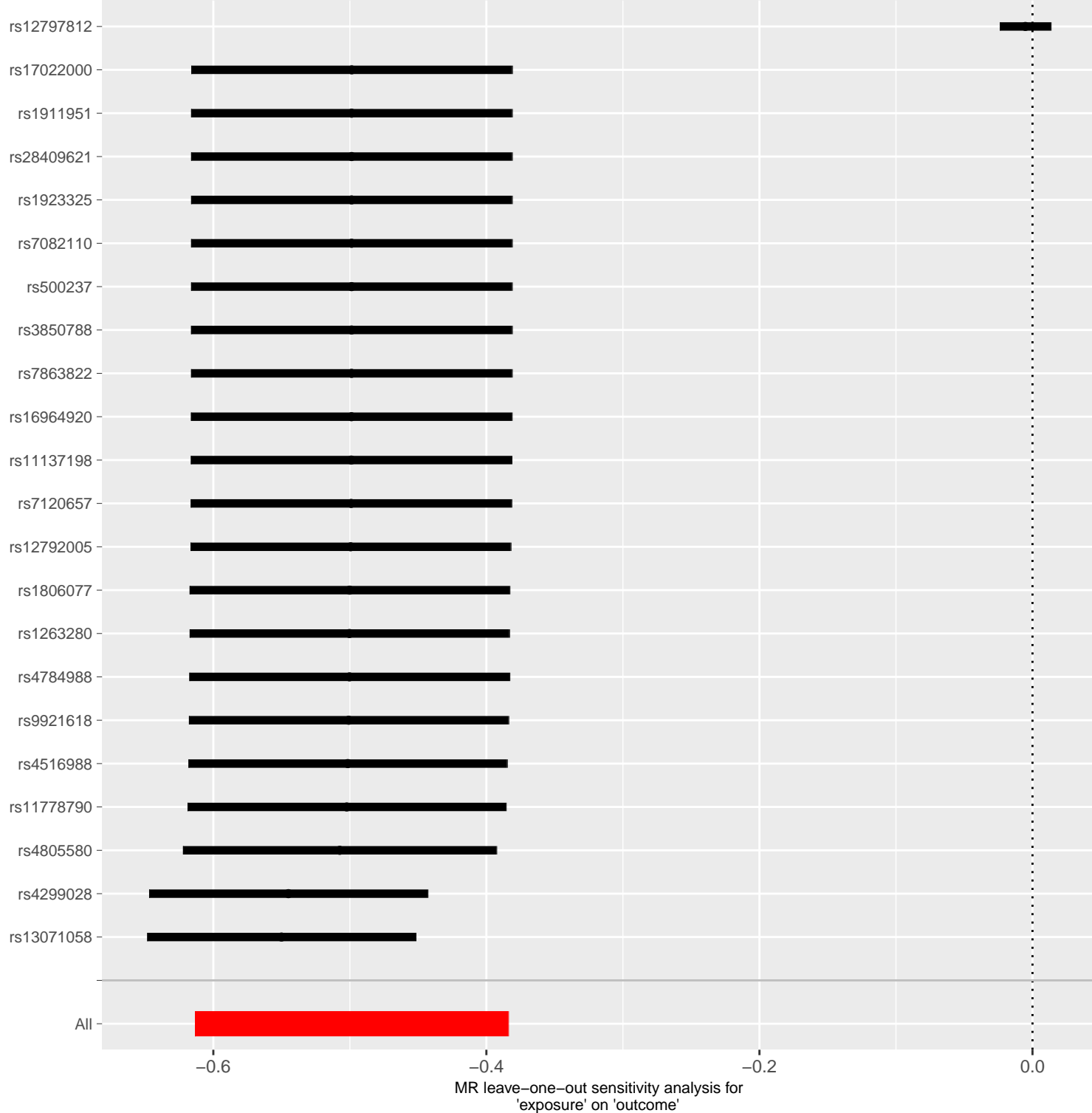

Supplement: Supplementary file 2 [file Datasheet1.zip › Data Sheet 1_v1/Supplementary Figures 1-10/2-hydroxyacetaminophen sulfate_sensitivity-analysis.pdf]

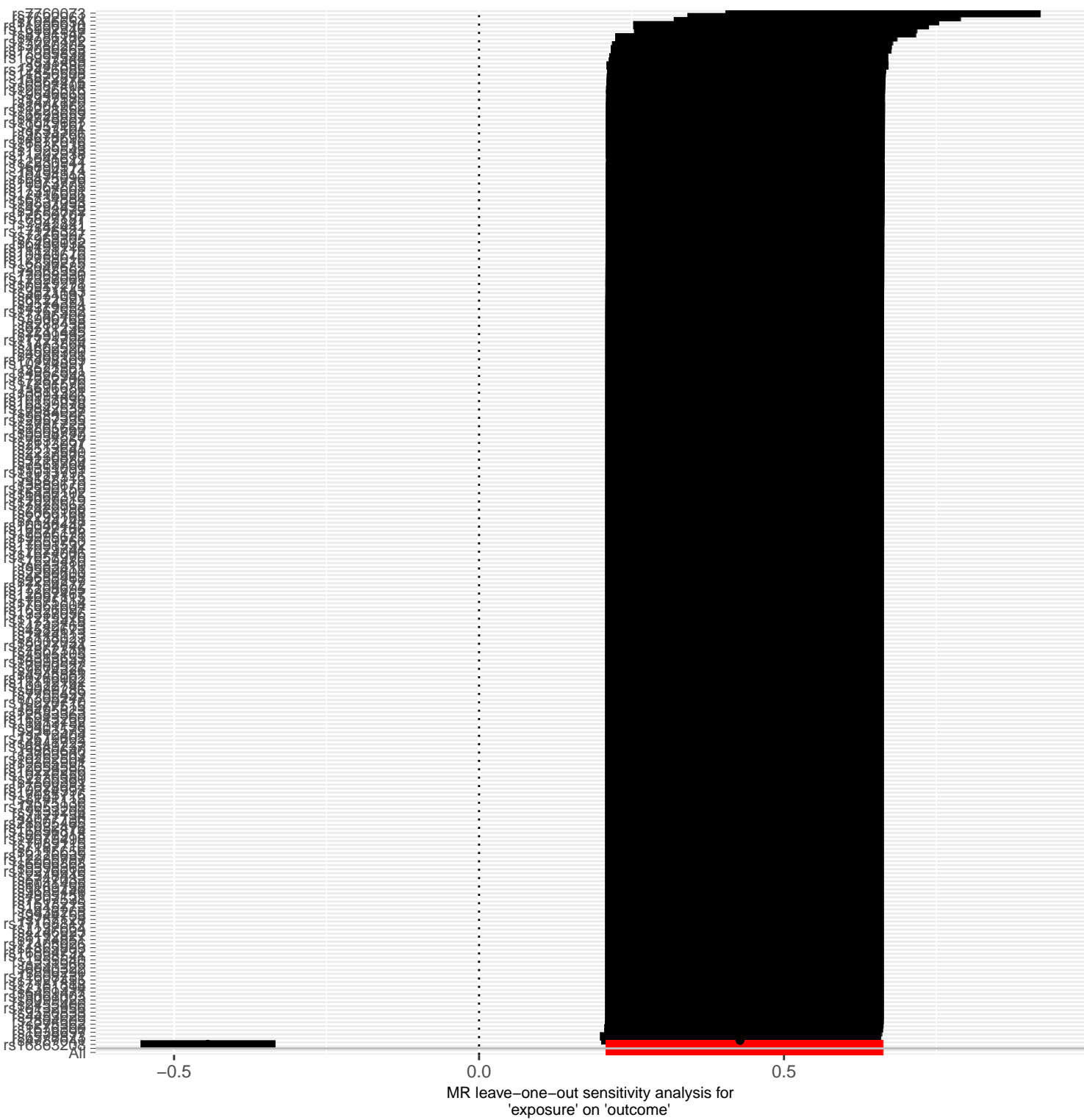

Supplement: Supplementary file 2 [file Datasheet1.zip › Data Sheet 1_v1/Supplementary Figures 1-10/2-methoxyacetaminophen sulfate_sensitivity-analysis.pdf]

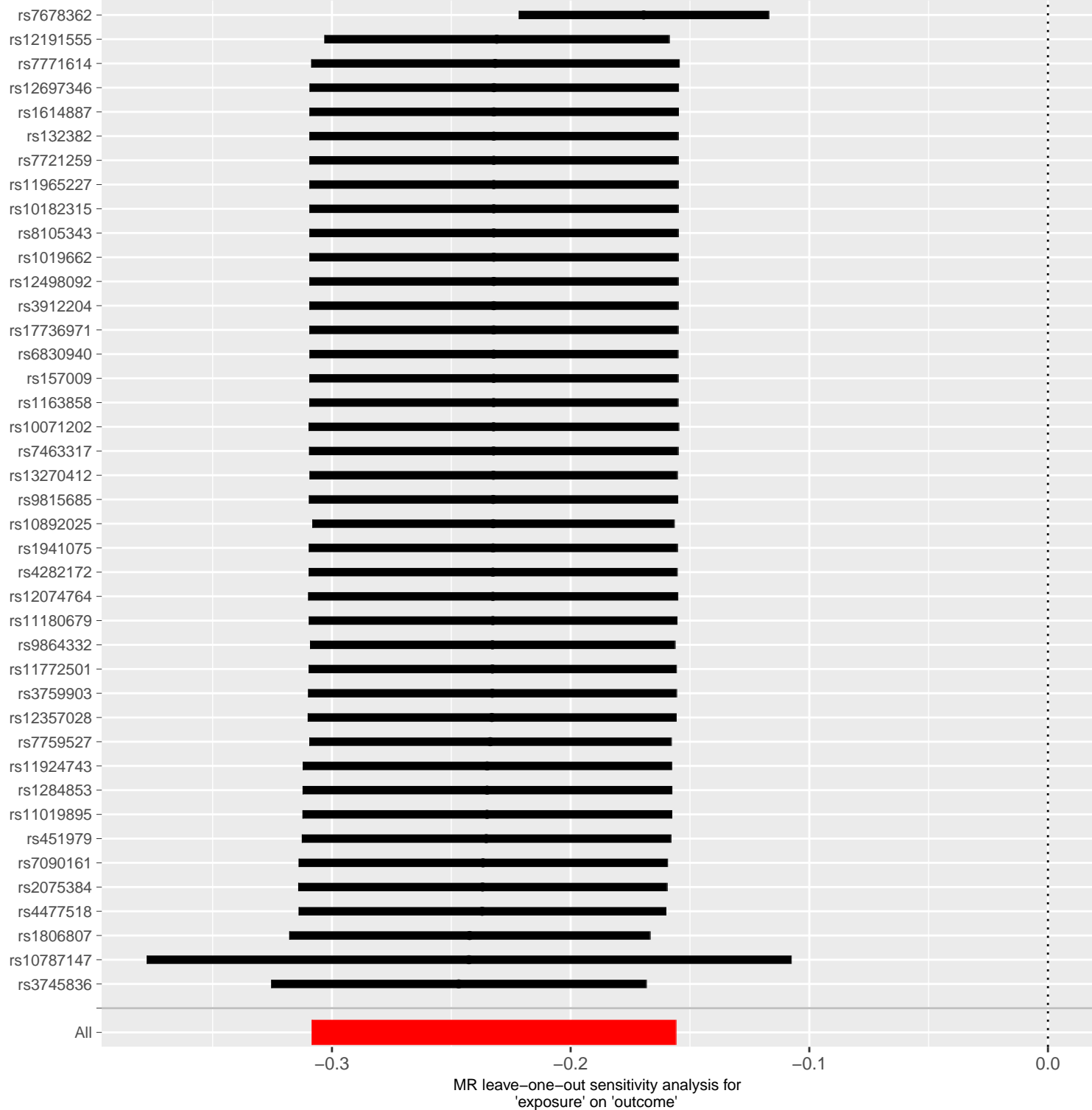

Supplement: Supplementary file 2 [file Datasheet1.zip › Data Sheet 1_v1/Supplementary Figures 1-10/3-(cystein-S-yl)acetaminophen_sensitivity-analysis.pdf]

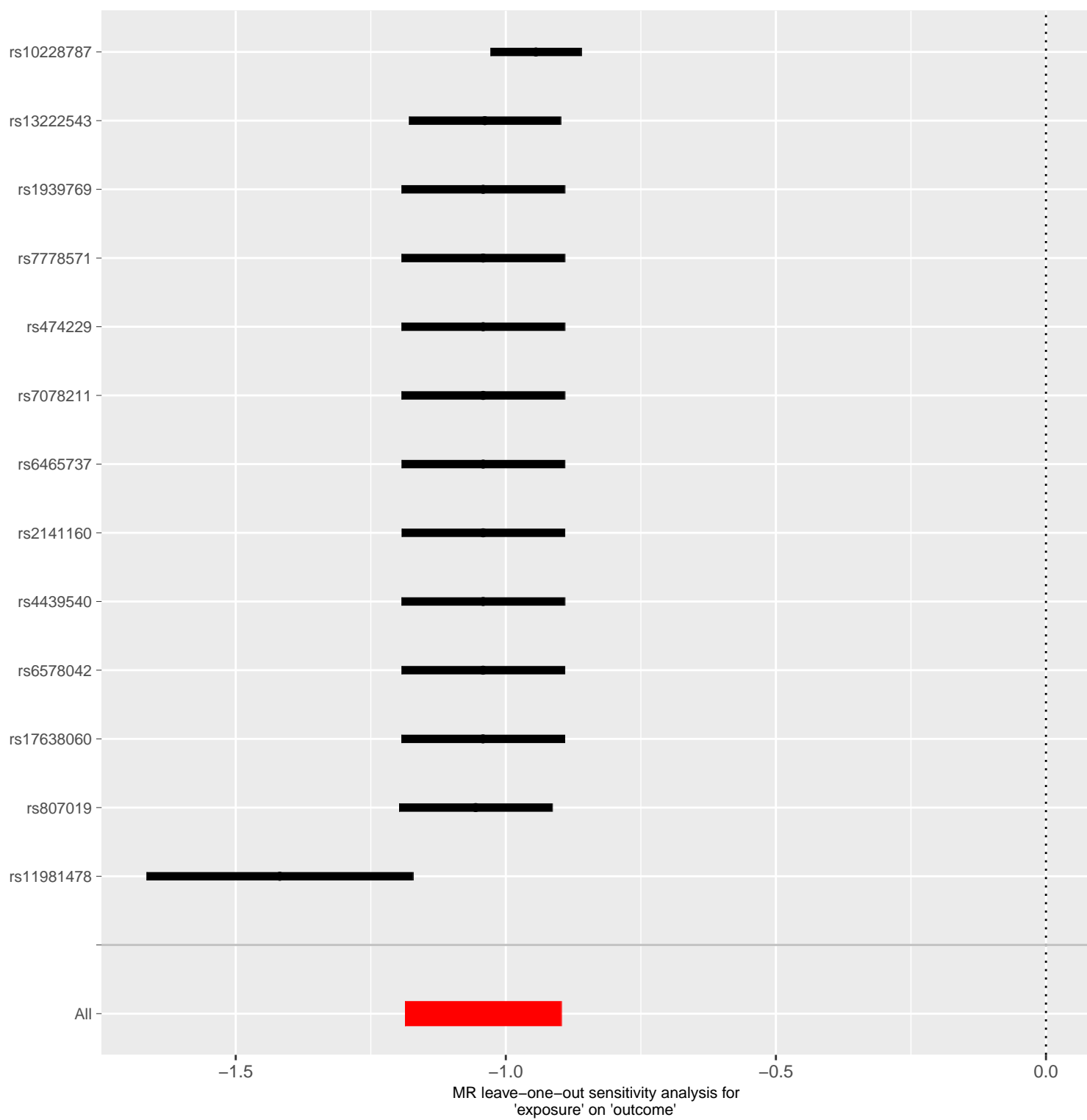

Supplement: Supplementary file 2 [file Datasheet1.zip › Data Sheet 1_v1/Supplementary Figures 1-10/epiandrosterone sulfate_sensitivity-analysis.pdf]

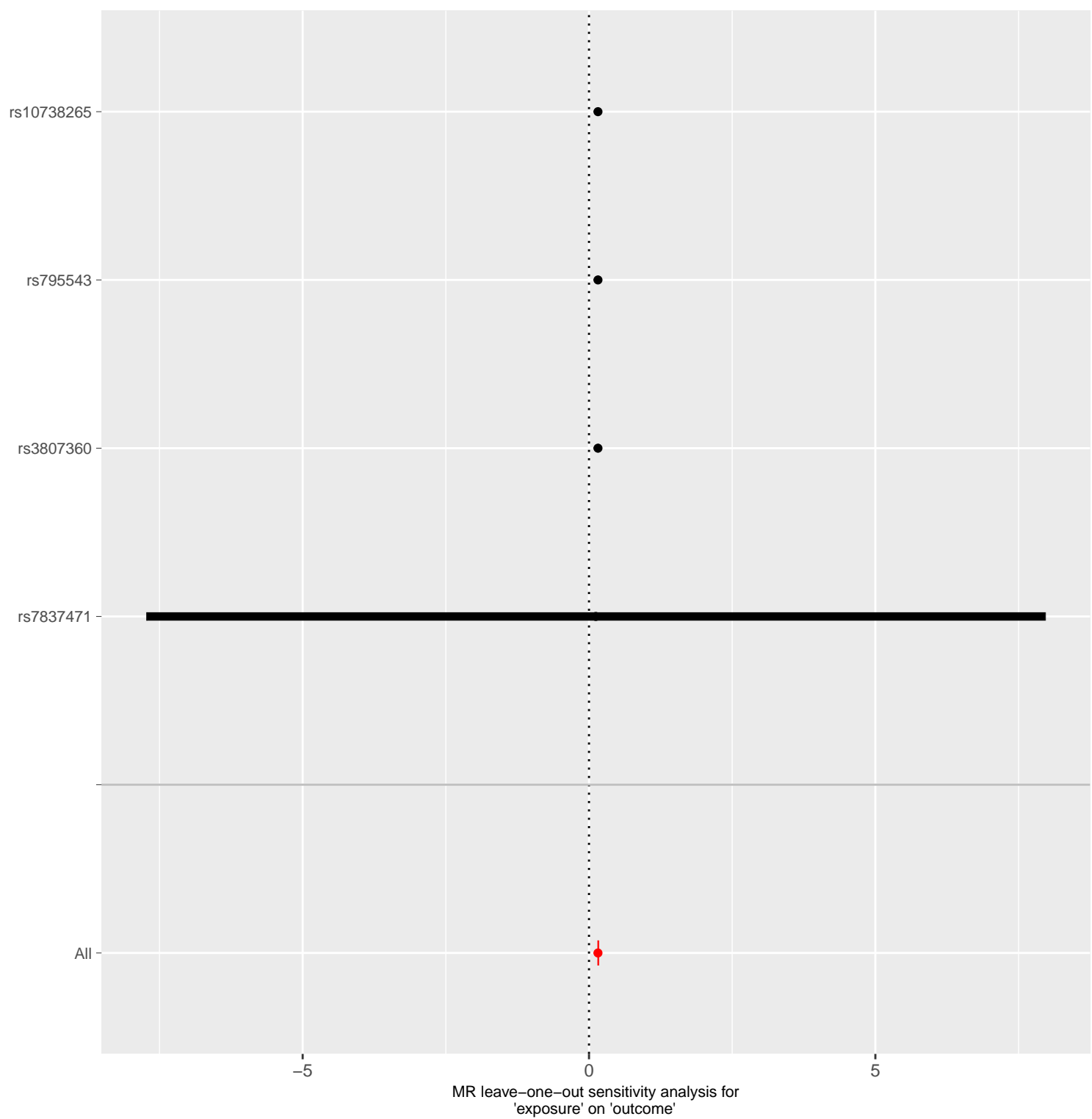

Supplement: Supplementary file 2 [file Datasheet1.zip › Data Sheet 1_v1/Supplementary Figures 1-10/glycocholate_sensitivity-analysis.pdf]

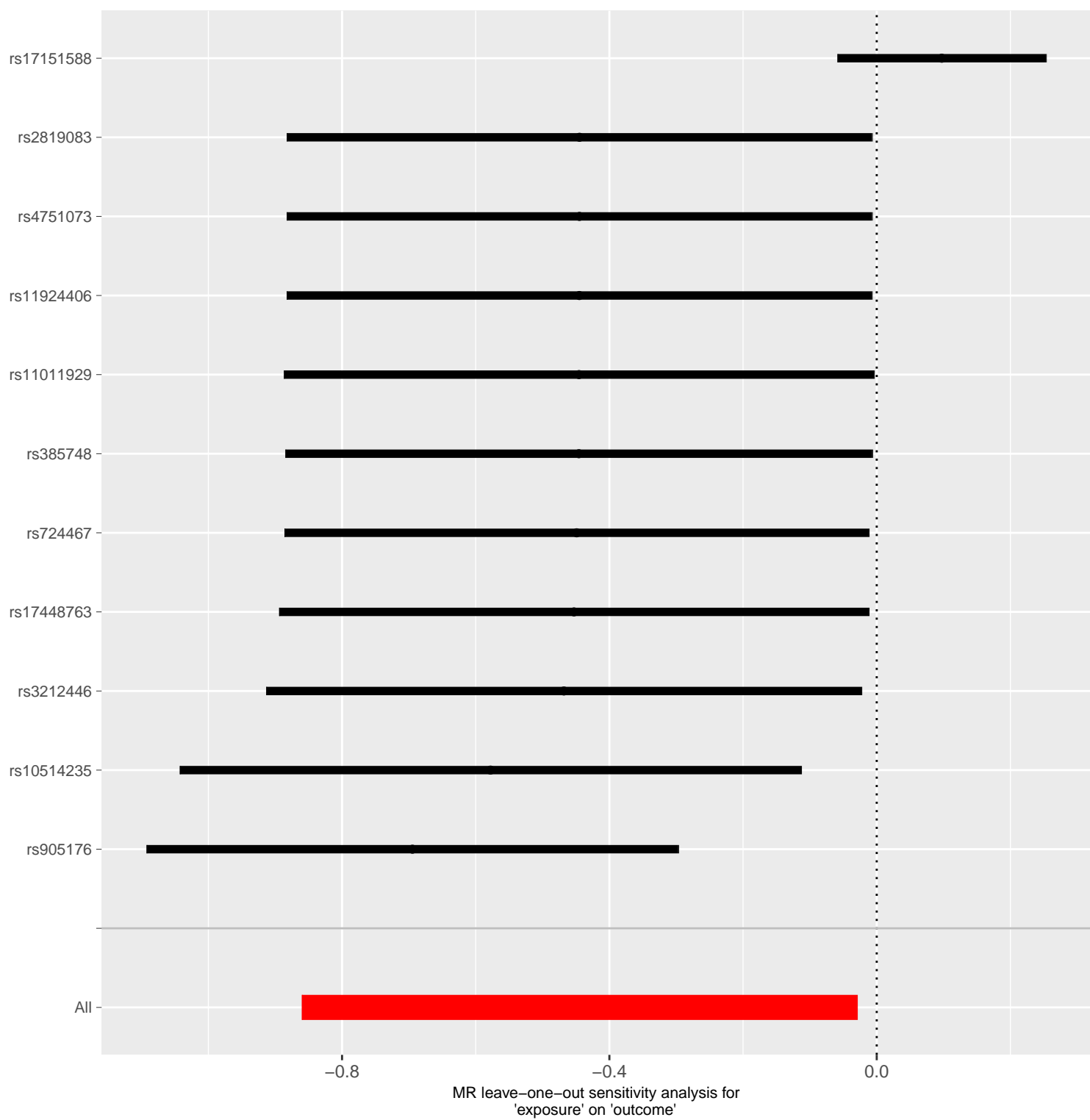

Supplement: Supplementary file 2 [file Datasheet1.zip › Data Sheet 1_v1/Supplementary Figures 1-10/hydroquinone sulfate_sensitivity-analysis.pdf]

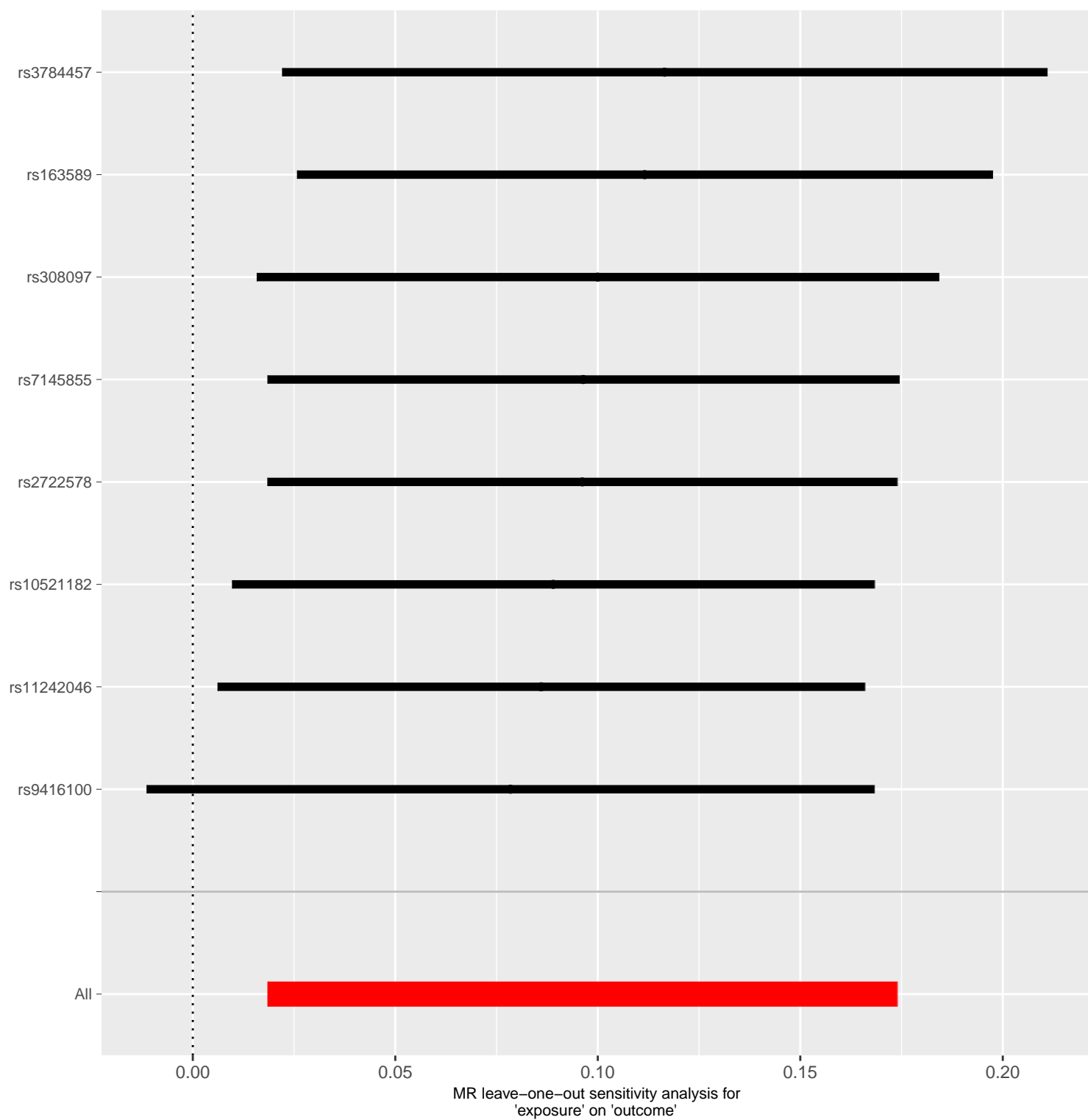

Supplement: Supplementary file 2 [file Datasheet1.zip › Data Sheet 1_v1/Supplementary Figures 1-10/salicyluric_sensitivity-analysis.pdf]

rs7342491

rs7429997

rs9863850

rs6926138

All

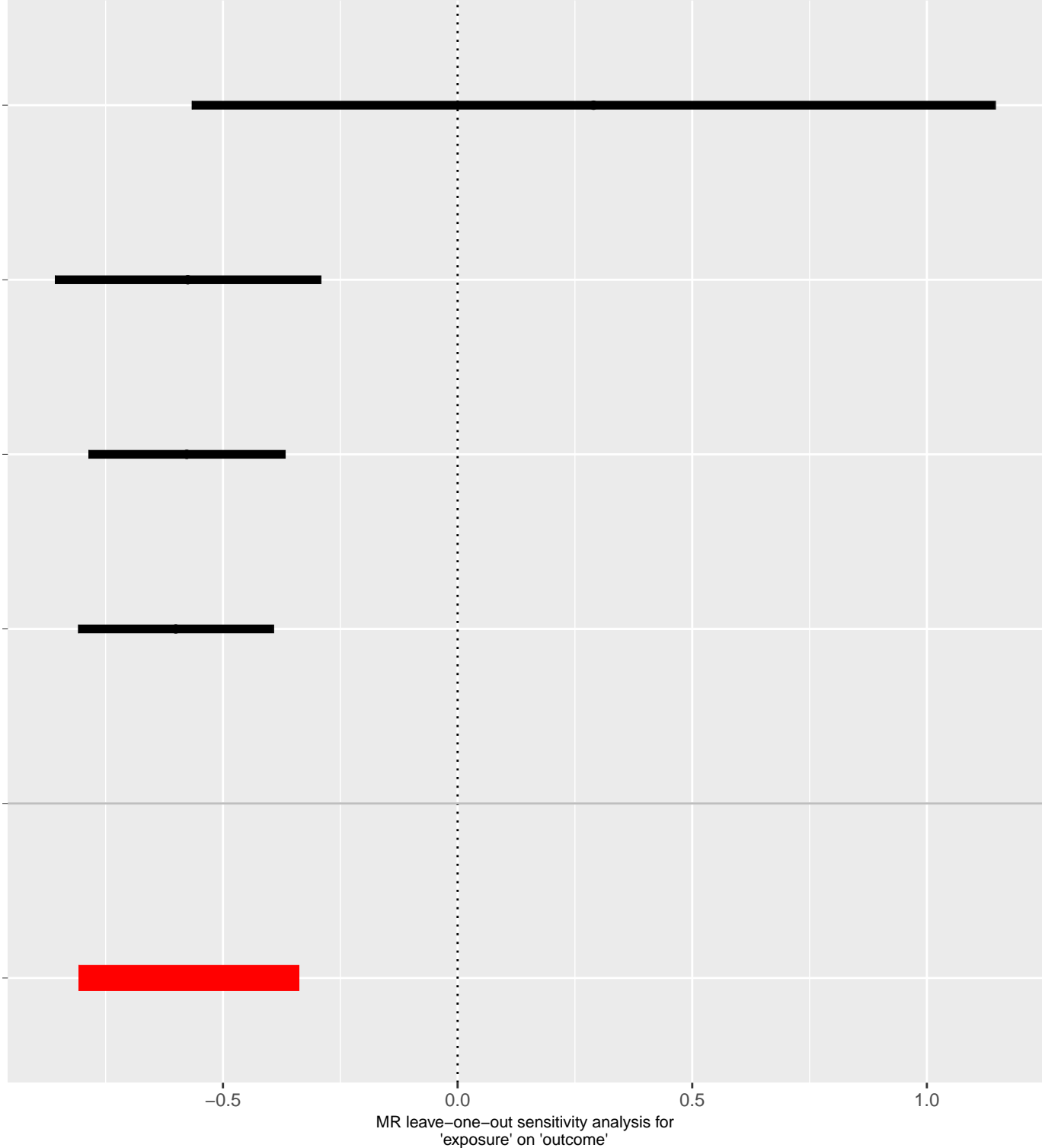

-0.5

0.0

0.5

1.0

MR leave-one-out sensitivity analysis for  
'exposure' on 'outcome'

Supplement: Supplementary file 2 [file Datasheet1.zip › Data Sheet 1_v1/Supplementary Figures 1-10/trans-4-hydroxyproline_sensitivity-analysis.pdf]

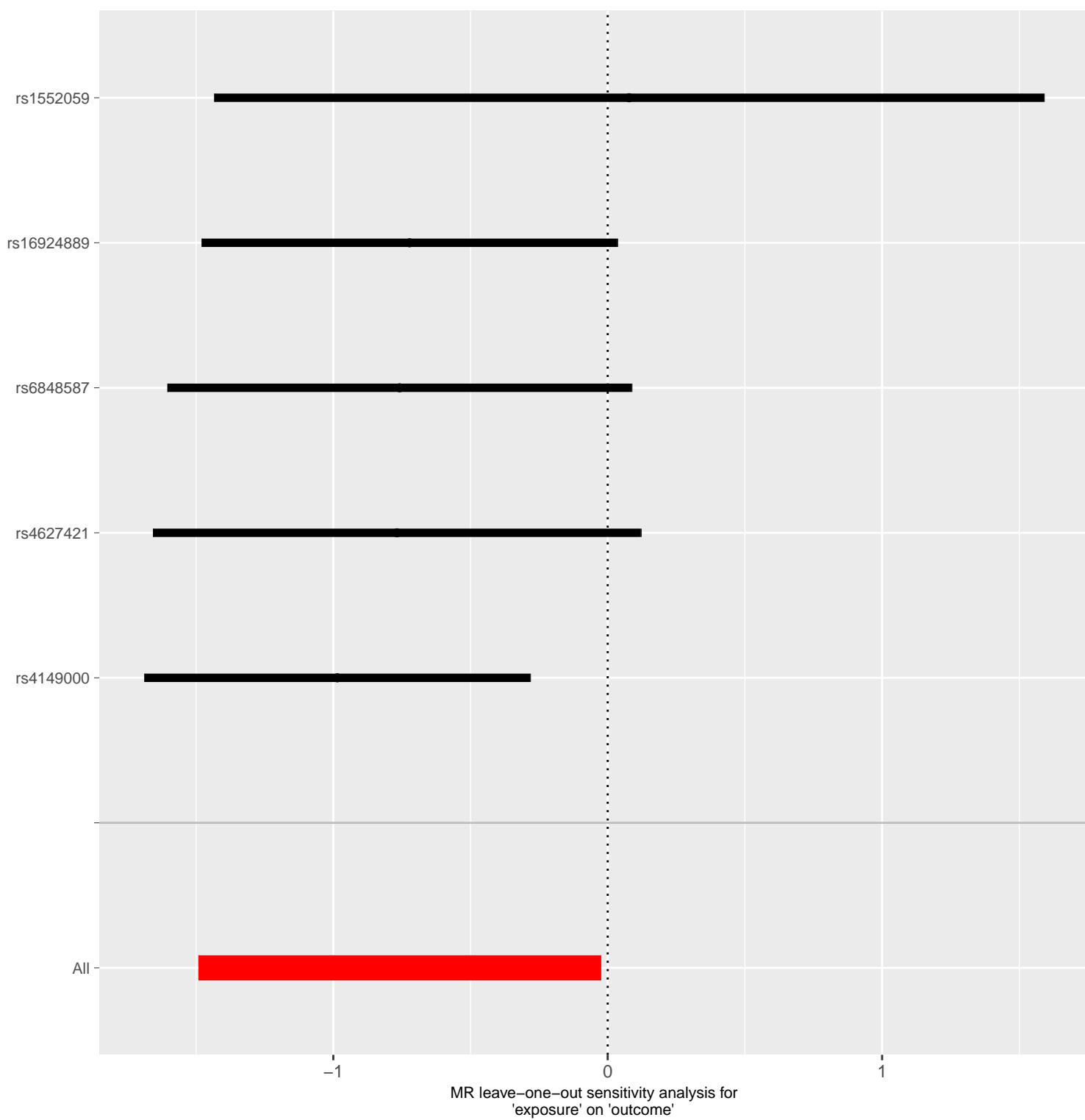

Supplement: Supplementary file 2 [file Datasheet1.zip › Data Sheet 1_v1/Supplementary Figures 1-10/Z-bilirubin_sensitivity-analysis.pdf]
